# Arabian Levantine viper bite induces thrombocytopenia – a case report

**DOI:** 10.25122/jml-2021-0283

**Published:** 2022-06

**Authors:** Ayman Abukamar, Rawan Abudalo, Mazen Odat, Mohammad Al-Sarayreh, Maher Bani Issa, Asem Momanie

**Affiliations:** 1 Department of Hematology and Oncology, King Hussein Medical City, Amman, Jordan; 2 Department of Clinical Pharmacy and Pharmacy Practice, Faculty of Pharmaceutical Sciences, The Hashemite University, Zarqa, Jordan; 3 Department of Neurology Unit, King Hussein Medical City, Amman, Jordan

**Keywords:** thrombocytopenia, disseminated intravascular complications (DIC), snake bite

## Abstract

Snakebites have been reported to induce hematological complications. Thrombocytopenia usually occurs secondary to disseminated intravascular coagulation (DIC) and coagulopathy induced by the snake bite. However, thrombocytopenia can develop after the snake bite, even in the absence of significant coagulopathy. We reported the case of a 36-year-old Jordanian male patient who was bitten by Arabian Macrovipera Lebetina Obtusa (Levantine viper), which developed venom-induced severe thrombocytopenia without coagulopathy. A progressive drop in platelet count was observed during his admission. His condition improved after anti-venom therapy, and he was discharged after 4 weeks of treatment for a full recovery. This case supports that snake venom can produce severe thrombocytopenia without significant coagulopathy, which can be treated successfully with anti-venom and the best supportive care.

## INTRODUCTION

Currently, about 3000 snake species are known worldwide. However, only 15% of these are venomous and, thus, dangerous to humans [1]. Males between 17 and 27 years are the most susceptible to snake bites with upper limbs as the usual site. Snake bites must always be taken seriously, and immediate medical attention is obligatory because these may induce death if not treated quickly and adequately [1–3].

Extreme age, improper handling of captive snakes (rather than wild encounters), delay in treatment, and insufficient treatment are risk factors that can cause death [4]. According to the World Health Organization (WHO), about 4.5 to 5.4 million snake bites occur each year worldwide, and 1.8 to 2.7 million of these cases develop illnesses [2]. It is estimated that at least 81,000 to 138,000 people die from snake bites each year [2].

The venom of a snake is complex, and it contains various toxins that are known to attack different organs in the body. While different species carry different types of venom, the major effects of venoms include the following [5]:

1. Cytotoxins lead to swelling and necrosis of the affected part.
2. Hemorrhage induces disseminated intravascular coagulation (DIC), low platelets, and disrupts the blood vessels.
3. Neurotoxins lead to flaccid paralysis of the victim.
4. Myotoxins break down muscles, which leads to renal failure and electrolyte imbalance, and may, in turn, lead to cardiac arrest.

Macrovipera lebetina is a medically important species of snake. Subspecies from the Levantine viper are capable of causing hematological manifestations and necrosis, as seen in the case reported here. This snake is found in North Africa, the Middle East, and the Far East and is defined by the WHO grading scale as a class II snake of medical significance [6–8].

Five subspecies are currently recognized for Macrovipera lebetina (ML), mentioned in Table 1 [9, 10].

**Table 1:** Subspecies of Macrovipera lebetina in the world.

Subspecies	Locations
**Macrovipera lebetina *obtusa***	Jordan, Syria, Lebanon, Iraq, Turkey, India, Pakistan (Kashmir), Caucasus, Azerbaijan, Dagestan, Iran and southern Afghanistan
**Macrovipera lebetina *lebetina***	Cyprus
**Macrovipera lebetina *cernovi***	Southern Turkmenistan, Pakistan (Kashmir) northern Afghanistan and Iran
**Macrovipera lebetina *transmediterranea***	Tunisia, Algeria
**Macrovipera lebetina *turanica***	Uzbekistan, Tajikistan, Eastern Turkmenistan, southwestern Kazakhstan, parts of western Pakistan and northern Afghanistan

**Figure 1:**
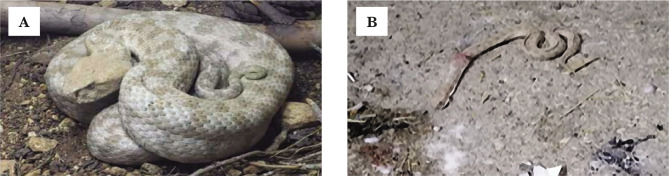
Arabian Macrovipera Lebetina Obtusa (Levantine viper). A – Live snake photo has been taken by the Royal Society for the Protection of Nature/Jordan. B – The snake killed by the patient.

Levantine viper (Macrovipera lebetina obtusa) is a large snake with a length that possibly exceeds 150 cm and a triangular head that is clearly distinct from the neck (Figure 1 A, B). Interestingly, there is a difference in size between the sexes as females are larger than males.

Belonging to the family of Viperidae, Levantine has a very limited distribution in the Rocky Mountains with low vegetation in Tafilah Governorate in southern Jordan [11].

The bite of Macrovipera lebetina obtusa can cause death. However, the mortality rate is unknown, as there are poor accurate statistics due to insufficient documented data from the countries where this snake is found. In addition to the local effects, *i.e*., pain, progressive edema, ecchymosis, and blisters, other systemic effects can occur, including nausea, vomiting, dyspnea, neurologic abnormalities, hypotension, shock, and hematological abnormalities. Viperidae envenomation is mostly known to cause coagulopathy disturbances, including mucosal and internal hemorrhages, thrombocytopenia, and a decrease in coagulation factors [12–14].

The venoms produced by the deadly Viperidae snake family contain hemotoxin and vasculotoxic components [13–15]. Hence, following their envenomation, coagulopathy disturbances, including spontaneous bleeding and thrombocytopenia, are predictable complications [16, 17]. Several hematological consequences can occur after a snake bite, including platelet aggregation and adenosine triphosphate (ATP) release by platelet aggregating proteins, platelet inactivation due to binding of venom antigens to platelet integrins, consumption of coagulation factors, and damage to blood vessels. Anti-venom administration can restore coagulopathy sufficiently and rapidly [16]. However, thrombocytopenia seems difficult to resolve, and the victim has 3.1 times the risk of difficulty in achieving the normal range of platelets [18].

Most patients who have been bitten by snakes received anti-venom administration during their admission to the hospitals. The main goal of the anti-venom administration is to obtain neutralization of the venom inside the body, thereby disabling its action and effects.

## CASE REPORT

**Figure 2:**
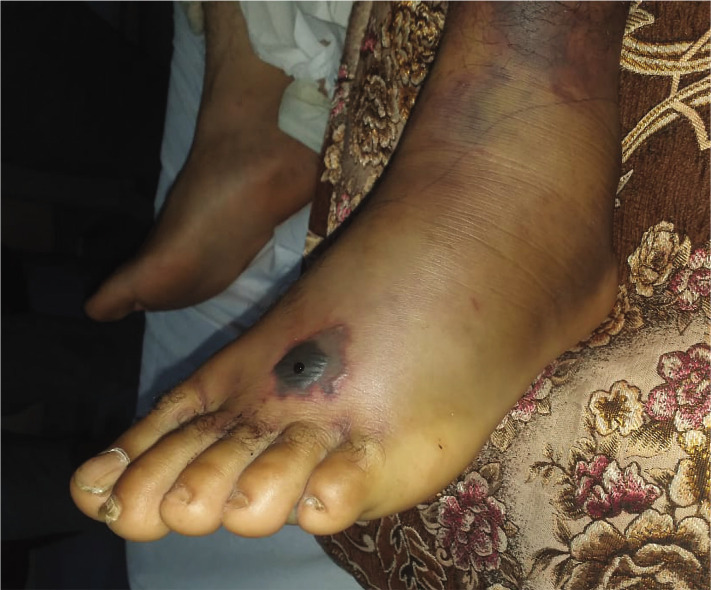
Patient's left foot after the snake bite. Swelling associated with discoloration was reported.

A 36-year-old male, who works as a farmer, presented to the emergency room at his local hospital after being bitten by a snake on the dorsum of the left foot while he was watering the field. He complained of pain in the left leg and dizziness associated with the swelling at the site of the snake bite. The pain was diffuse, burning in nature. It was ascertained through clinical examination that his vital signs were stable, and his left foot and ankle joint was swollen and hot. Later, a black discoloration developed at the site of the bite (Figure 2) with ecchymosis. A lab test immediately revealed the following: White blood cells (WBCs) count was 7.5×10^3^/µL, 68% neutrophils, hemoglobin was 11.5 g/dl, platelets count was 114×10^3^/µL, and the international normalized ratio (INR) was 1.1 with normal prothrombin time (PT), partial thromboplastin time (PTT), and kidney function test (KFT). After hospitalization, supportive treatment with IV fluids and IV antibiotics was started immediately in addition to anti-venom administration. In the lab investigations repeated 6 hours later, the INR remained normal, his hemoglobin was 10.1 g/dl, and the platelets count was103×10^3^/µL. However, with subsequent days, his platelet count dropped more and more without significant coagulopathy with a progressive increase in the swelling of the left leg. He was referred to the King Hussein Medical City (KHMC), a tertiary hospital in Amman/Jordan.

**Figure 3:**
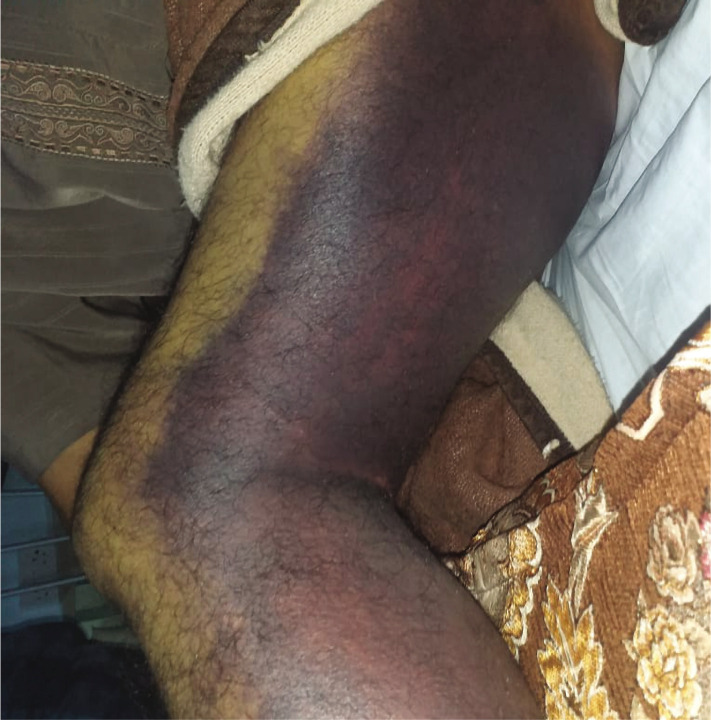
Representative image of patient's left leg with diffuse subcutaneous hemorrhage, which involved the whole limb.

Upon arrival, his vital signs were stable, he was fully conscious, and there was no neurological deficiency. However, his left leg was swollen with ecchymosis, and diffuse subcutaneous bleeding was reported on examination (Figure 3). Further laboratory tests were repeated, which revealed the following: hemoglobin level was 9.8g/dl, platelets count was 99.7×10^3^/µL, INR was 1.24, WBCs count was 7.57×10^3^/µL, and normal KFT. After hospitalization in our hospital, he received the best supportive care, but a progressive drop in the platelet count was observed each subsequent day until it reached 2×10^3^/µL. Blood film and manual platelet count revealed neutrophilic leukocytosis and normochromic normocytic anemia, and the manual count for the aggregated platelets with thrombocytopenia was 50000×10^3^/µL. The patient received the best supportive care until full recovery and was discharged from the hospital after 5 weeks with adequate platelets count. His laboratory results during his stay in the hospital were summarized in Table 2.

**Table 2:** Patient's laboratory results after the snake bite.

Test	D1	D2	D3	D4	D5	D10	D15	D20	D25	D30
**WBCs**	7.5	8.1	7.57	20.0	13.34	12.75	17.49	12.02	10.6	10.3
**Hb**	11.5	10.1	9.8	11.3	10.9	10.8	11	11.8	13.4	12.2
**Platelets**	114	103	99.7	41	6	2	9	6	14.1	145
**INR**	1.13	1.29	1.24	1.06	1.14	1.12	0.97	1.02	1.1	0.97
**PT**	12.2	17.2	16.5	14.2	15.2	15	13.2	13.8	14.5	14.1
**PTT**	21.6	27.6	25.8	24.8	25	25.1	24.8	25	27.1	26.3
**Creatinine**	0.6	0.9	0.85	0.9	1.0	1.1	0.9	0.8	1.0	0.9

Lab tests were done at different points in time; day 1 (D1), day 2 (D2), day 5 (D5), day 6 (D6), day 7 (D7), day 10 (D10), Day 15 (D15), day 20 (D20), day 25 (D25), and day 30 (D30).

## DISCUSSION

The toxic component found in the venom of any snake leads to various pathological consequences ranging from local, such as pain, swelling, or bruising, to systemic consequences, which include neurotoxicity, myotoxic, and hematological toxicities. The severity of these consequences depends on the amount of venom and site of the bite. The hematological consequences of a snake bite are several, including DIC, hemolysis, thrombocytopenia, and bleeding due to consumption of clotting factors. Both development of DIC and an increase in vascular permeability may be observed. Bleeding ranged from subcutaneous to severe such as hematuria and hemoptysis. Indeed, thrombocytopenia can occur after a snake bite without significant coagulopathy, as discussed in our case. As such, the proposed mechanisms of thrombocytopenia are related to the increase of platelet aggregations induced by the venom or anti-venom or due to direct destruction of the platelets since another mechanism may coexist. Platelet transfusion is eventually required if severe thrombocytopenia or bleeding to major organs has occurred.

Thrombocytopenia usually improved with anti-venom administration and supportive treatment for most patients, but sometimes, prolonged periods were required for complete recovery.

## CONCLUSION

The reported case supports the hypothesis that thrombocytopenia can occur without coagulopathy after a snake bite.
